# Can artificial intelligence identify effective COVID‐19 therapies?

**DOI:** 10.15252/emmm.202012817

**Published:** 2020-07-07

**Authors:** Michael B Schultz, Daniel Vera, David A Sinclair

**Affiliations:** ^1^ Paul F. Glenn Center for Biology of Aging Research Blavatnik Institute Harvard Medical School Boston MA USA

**Keywords:** Microbiology, Virology & Host Pathogen Interaction, Pharmacology & Drug Discovery

## Abstract

In this issue of *EMBO Molecular Medicine*, Stebbing *et al* (2020b) validate an artificial intelligence‐assisted prediction that a drug used to treat rheumatoid arthritis could be a potent weapon against COVID‐19. Using liver organoids infected with SARS‐CoV‐2, they confirm dual antiviral and anti‐inflammatory activities and show that its administration in four COVID‐19 patients is correlated with disease improvement, paving the way for more rigorous placebo‐controlled trials.

## Graphical abstract

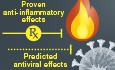

Severe COVID‐19 cases are associated with hyperactivation of the immune system, which can lead to acute respiratory distress syndrome, other secondary immune disorders, and death (Mehta *et al*, 2020). Antivirals can help slow the course of an infection but typically do not mitigate hyperactive immune responses. Similarly, immunosuppressants used to limit uncontrolled inflammation may also limit a patient's capacity to clear the virus. Therefore, a drug that combines antiviral and immunosuppressant activities could be a powerful treatment (Perricone *et al*, 2020). In this issue of *EMBO Molecular Medicine*, Stebbing *et al* (2020b) test the prediction that an anti‐inflammatory molecule used for rheumatoid arthritis (RA) also has antiviral activity and could improve the outcomes of those afflicted by severe COVID‐19 (Fig 1).

**Figure 1:**
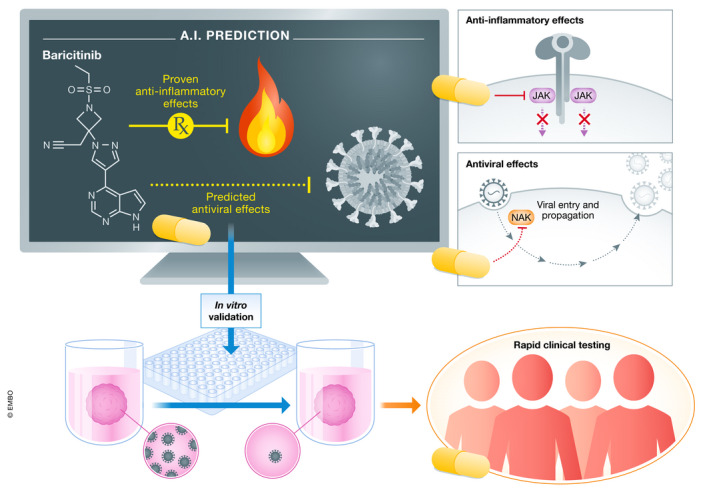
Baricitinib's journey as a candidate therapeutic for severe COVID‐19 An artificial intelligence platform called BenevolentAI identified baricitinib, a Janus kinase (JAK) inhibitor used to treat rheumatoid arthritis, as a potential COVID‐19 therapy. The platform predicted it might also inhibit Numb‐associated kinases (NAKs), which are important for viral entry into a cell. In their new work, Stebbing and colleagues validate these properties in *in vitro* models and provide evidence from four patients that baricitinib may be beneficial in COVID‐19 patients, prompting several placebo‐controlled clinical trials that are now underway.

A typical drug development program takes at least 5 years to move from bench to bedside. This timeframe can be shortened to months by repurposing existing drugs. The question is, how to rapidly identify which existing drugs to test in patients? A possible breakthrough was made in February by Stebbing and colleagues, a London‐based team, who used an artificial intelligence platform called BenevolentAI to identify baricitinib as a possible COVID‐19 therapy (Richardson *et al*, 2020; Stebbing *et al*, 2020a). While there are few specific details available how BenevolentAI works, conceptually similar platforms are increasingly being used to generate novel hypotheses that are speeding drug repurposing efforts (Park, 2019).

Baricitinib is an orally available, small molecule inhibitor of Janus kinase (JAK) that has anti‐inflammatory properties (Schwartz *et al*, 2017). It is approved in both Europe and the United States as a second‐line therapy for RA and is in clinical trials for other autoimmune diseases such as lupus, graft‐versus‐host disease, and psoriasis (clinicaltrials.gov). BenevolentAI suggested that the molecule may also have antiviral properties, based on its predicted ability to inhibit two members of Numb‐associated kinase (NAK) family, AAK1 and GAK. NAKs mediate the trafficking of vesicles from the cell surface to endosomes, a key step in the viral entry process after receptor binding (Sorrell *et al*, 2016; Schor & Einav, 2018). If these predictions are correct, then baricitinib could serve as an antiviral in the early stages of COVID‐19 and also prevent cytokine storms in the later stages of the disease.

To test these predictions, the authors first confirmed baricitinib's broad anti‐inflammatory properties against an array of immune agonists using human monocytes, NK cells, and CD4 and CD8 T cells. Baricitinib also suppressed levels of IL‐6 in RA patients, a cytokine that is elevated in COVID‐19. They then tested whether baricitinib indeed has antiviral activity. As predicted, baricitinib bound to multiple members of NAK enzymes at physiologically relevant concentrations. In human liver spheroids infected with live SARS‐CoV‐2, baricitinib reduced viral load by more than 30%, strongly supporting the hypothesized antiviral activity.

With this promising *in vitro* data in hand, the authors sought to test baricitinib in patients with severe COVID‐19. As baricitinib is already approved for RA, the authors received permission for its compassionate use in COVID‐19 patients with bilateral pneumonia. Four patients were selected, many with significant comorbidities, between the ages of 29 and 76. One patient was receiving continuous positive airway pressure to assist in his breathing. All four had elevated plasma IL‐6 and C‐reactive protein levels, demonstrating systemic inflammation. While only limited conclusions can be inferred from the experiences of just a few patients with no control subjects, these early results warrant further investigation. Within days of starting baricitinib treatment, the patients’ fevers and coughs improved, along with decreased viral loads and IL‐6 levels. Transient increases in levels of liver enzymes ALT and AST were also observed in the serum, suggesting possible mild liver toxicity, but no serious adverse events were noted.

These initial case reports have prompted the initiation of larger, better controlled safety and efficacy studies. The largest of these is a randomized, double‐blind, placebo‐controlled trial sponsored by the United States’ National Institute of Allergy and Infectious Diseases, enrolling over 1,000 patients. The trial, called ACTT2, will evaluate whether baricitinib in combination with the antiviral ribonucleotide analog remdesivir speeds up time to recovery compared to remdesivir treatment alone. Nearly a dozen smaller trials in Europe and the United States have also been initiated, alone or in combination with other drugs (clinicaltrials.gov). With these trials, baricitinib joins a small set of other anti‐inflammatory drugs, such as the anti‐IL‐6 and IL‐6R monoclonal antibodies tocilizumab and sarilumab, and the corticosteroid dexamethasone, as a leading candidate therapy for severe COVID‐19 (Cox, 2020).

Beyond these immediate next steps, the study also has broader, longer‐term implications. Other deadly viruses, such as those that cause Ebola, hepatitis C, dengue fever, and other coronavirus diseases, rely on similar mechanisms for entry into host cells and also cause systemic inflammation. Baricitinib, or drugs like it, could therefore become part of humanity's arsenal against emerging infectious diseases (Bekerman *et al*, 2017). Given the rush to develop novel anti‐COVID‐19 therapies, the authors did not test baricitinib in an animal model of viral infection, but such experiments could help determine whether antiviral NAK inhibition, anti‐inflammatory JAK inhibition, or both are necessary for the drug's putative efficacy. If baricitinib indeed does show efficacy via both mechanisms, it would validate NAKs as *bona fide* drug targets and elevate the importance of discovering other drugs that provide dual antiviral and anti‐inflammatory properties.

As the COVID‐19 pandemic is demonstrating, adversity fosters innovation. Here, the marriage of machine learning and rapid clinical trials provides hope for progress, not only in today's fight against COVID‐19, but in the ongoing fight against acute and chronic diseases.

## Conflict of interest

DAS is a cofounder, shareholder and consultant to EdenRoc companies, Life Biosciences companies, Galilei, Alterity, InsideTracker. Metrobiotech, an EdenRoc company, is developing NAD boosting molecules for age‐related diseases and COVID‐19. Other activities are listed at https://genetics.med.harvard.edu/sinclair-test/people/sinclair-other.php.
